# Relations between tension-type headache, sleep problems and temporomandibular pain?

**DOI:** 10.1186/1129-2377-14-S1-P53

**Published:** 2013-02-21

**Authors:** N Caspersen, JR Hirsvang, L Kroell, F Jadidi, L Baad-Hansen, P Svensson, R Jensen

**Affiliations:** 1 Danish Headache Center, Department of Neurology, Glostrup Hospital,University of Copenhagen, Denmark; 2 Section of Clinical Oral Physiology, Departement of Dentistry, University of Aarhus, Denmark

## Introduction

Tension-type headache (TTH) is the most prevalent headache among adults and is associated with impaired functional and psychosocial quality of life. Further analysis of eventual pathophysiology and comorbidities is needed. Temporomandibular Disorders (TMD) and TTH are frequently coexisting disorders and both characterized by increased pericranial tenderness. In a specialised tertiary headache centre more than half of the patients were also diagnosed with TMD but their eventual causality is yet unknown. Likewise is the relationship between tenderness, sleep quality and oral health in TTH patients also unknown. Our aim was to characterize the relationship between pericranial tenderness, sleep quality and oral health in TTH patients in comparison with healthy controls.

## Material and methods

The survey included 58 consecutive patients with frequent, episodic TTH or chronic TTH from a tertiary Headache Center and 58 healthy controls. Questionnaires regarding The Research Diagnostic Criteria for Temporomandibular Disorders (RDC), Oral Health Impact profile and Sleep/tiredness/snoring were completed.

## Results

TTH-patients were significantly more affected in the RDC-Screening by increased characteristic pain intensity (CPI) regarding self reported temporomandibular pain (TMP) (p<0.001), decreased quality of life (p<0,001), and the total sleep score (p<0.001) compared to healthy controls).

## Conclusion

TTH patients are severely affected by TMP; demonstrate impaired sleep quality and oral health function and may reflect common underlying pathophysiological mechanisms.
